# Speech recognition thresholds correlate with tinnitus intensity in individuals with primary subjective tinnitus

**DOI:** 10.3389/fneur.2025.1672762

**Published:** 2025-08-29

**Authors:** Panayiota Mavrogeni, Stefani Maihoub, András Molnár

**Affiliations:** ^1^Faculty of Health Sciences Doctoral School, University of Pécs, Pécs, Hungary; ^2^Maihoub ENT Clinic, Limassol, Cyprus; ^3^Opera Clinic, Budapest, Hungary

**Keywords:** tinnitus, tinnitus intensity, pure-tone audiometry, tinnitus matching, tinnitus severity, speech audiometry

## Abstract

**Objectives:**

This study aimed to analyze the relationship between tinnitus and speech audiometry results.

**Materials and methods:**

In this investigation, 314 patients with primary subjective tinnitus and 279 patients with sensorineural hearing loss, serving as a control group, were enrolled. All participants underwent comprehensive assessments, which included pure-tone and speech audiometry.

**Results:**

In considering basic parameters, a slight predominance of females was noted in both groups, with left-sided and bilateral tinnitus being the most common types. There were no significant differences in pure-tone averages between the tinnitus and control groups. In the tinnitus group, speech audiometry intensity were significantly higher (*p* < 0.00001) compared to the control group. In analyzing the relationships between tinnitus intensities and speech audiometry intensity, a significant (*p* = 0.000) positive correlation (rho = 0.581) was revealed. Additionally, a significant (*p* = 0.027) positive correlation (rho = 0.227) was found between tinnitus intensities and Tinnitus Handicap Inventory scores.

**Conclusion:**

The relationship between tinnitus intensities and speech audiometry intensity demonstrates how tinnitus affects speech comprehension. Additionally, the intensity of tinnitus significantly influences an individual’s perception of tinnitus severity.

## Introduction

Tinnitus is the perception of sounds without any external sound source (1). The prevalence of tinnitus is increasing globally, significantly impacting quality of life and leading to psychiatric symptoms (2). Tinnitus can be categorized into primary and secondary cases, depending on whether a specific underlying cause can be identified. Tinnitus can be classified as either subjective or objective, depending on whether the sounds can only be heard by the individual experiencing them (3). Primary cases typically involve sensorineural hearing loss, a prevalent cause of tinnitus (4). This condition affects the hearing nerve and the hair cells in the inner ear, which consist of both outer hair cells (OHCs) and inner hair cells (IHCs). While other ear-related issues such as otosclerosis, middle ear infections, and Ménière’s disease are commonly associated with tinnitus, it is also important to consider systemic causes (5). Persistent tinnitus that follows hearing loss is associated with changes in the central nervous system, resulting from plastic changes in central auditory structures (6). Previous studies suggest that this is related to the deafferentation of structures in the central nervous system. However, the specific type of inner ear damage that triggers these changes remains unknown (7). The role of central nervous system changes in tinnitus perception is underscored by the fact that tinnitus continues even after auditory nerve section or cochlear ablation (8). However, not all patients with hearing loss experience tinnitus, the causes of which remain unclear. Tinnitus can occur even in people with normal pure-tone audiometry results (9). This may be due to factors such as reduced responses from the cochlear nerve, weaker reflexes in the middle ear muscles, stronger medial olivocochlear efferent reflexes, and heightened activity in the central auditory pathways (10). Additionally, hearing loss in frequencies above 8,000 Hz can also explain normal audiometry results in individuals experiencing tinnitus. According to recent results, tinnitus, when combined with hearing loss, significantly raises the risk of depression. Therefore, it is crucial to screen for hearing loss (11). In everyday audiological practice, pure-tone audiometry is the most widely used method, including for patients with tinnitus. However, because this test only uses pure tones, it does not provide information on speech understanding, which is essential for daily life (12). Therefore, incorporating speech audiometry is crucial.

Given that patients with tinnitus frequently report difficulties in understanding speech, especially in cases of unilateral tinnitus compared to the unaffected ear, this study aimed to evaluate the potential influence of tinnitus on speech audiometry outcomes.

## Materials and methods

### Study population

A total of 314 patients with primary subjective persistent tinnitus and 279 patients with sensorineural hearing loss were enrolled in this study. The latter group served as the control group. All patients underwent thorough otorhinolaryngological and audiological assessments conducted by specialists experienced in managing tinnitus and hearing loss. The inclusion criteria for the study group consisted of patients over 18 years of age with subjective primary tinnitus, encompassing both acute and chronic cases. All participants provided written informed consent to take part in the investigation, and their clinical data were made available for analysis. The exclusion criteria were as follows: secondary cases of tinnitus (such as those caused by earwax buildup, otosclerosis, Ménière’s disease, or acoustic neuroma); any disorders that could affect the results of subjective hearing testing, including neurological and psychiatric conditions; internal medical diseases (e.g., hypertension or diabetes mellitus); endocrinological disorders; autoimmune disorders; significant stenosis of the carotid and vertebral arteries; use of ototoxic medication; and any history of treated malignancies involving systemic oncological treatment, etc. All patients underwent carotid and vertebral ultrasonography, as well as a contrast-enhanced brain MRI. A comprehensive audiological assessment was conducted, as detailed below. The basic clinical data of the participants are presented in Table 1. All participants provided written consent to participate in the study. The investigation adhered to the Declaration of Helsinki and received approval from the Hungarian ETT TUKEB (approval number: BM/29864–1/2024, approval date: December 9, 2024).

**Table 1:** Participants’ basic parameters.

Parameters	Tinnitus group (*n* = 314)	Control group (*n* = 279)	*p*-value
Age (median years; IQR, Q1–Q3)	54; 15.25 (47–62.25)	54; 20.5 (41.25–61.75)	*p* = 0.05*
Sex (men/women)	126/188	120/159	*p* = 0.47**
Pure-tone average			
Right (median dB; IQR, Q1–Q3)	40; 30 (15–45)	40; 25 (30–55)	*p* = 0.06*
Left (median dB; IQR, Q1–Q3)	40; 30 (15–45)	40; 30 (20–50)	*p* = 0.05*
Tinnitus location, *n* (%)			
Right	65 (20.7%)		
Left	91 (28.9%)		
Bilateral	158 (50.4%)		
Tinnitus onset (median months; IQR, Q1–Q3)	12; 31 (5–36)		

*Mann–Whitney *U* test, **Chi-squared test. The significance level was set at *p* < 0.05. IQR, interquartile range; Q1, first quartile; Q3, third quartile.

### Audiological examinations

Before the audiological examinations, all patients underwent microotoscopy and tympanometry, including acoustic reflex testing, to rule out potential causes of conductive hearing loss. Pure-tone audiometry was conducted using a GSI 61 Audiometer (Grason Stadler, Inc., Milford, CT, United States) by a qualified audiological assistant in each case. The examinations took place in a soundproof booth. Both air conduction (125–8,000 Hz) and bone conduction (250–4,000 Hz) were measured, utilizing headphones and a mastoid vibrator, respectively. In necessary cases, masked bone conduction measurements were performed. The lowest perceivable intensities were identified in 5 dB increments. Prior to the examinations, the sound stimuli were demonstrated to the patients to ensure they understood what to listen for. Standard manual audiograms were created, and pure-tone averages were calculated. Sensorineural hearing loss was defined based on the 1995 recommendations from the Committee on Hearing and Equilibrium of the American Academy of Otolaryngology–Head and Neck Surgery (13).

Tinnitus pitch and intensity matching were performed for each case. The pitch matching process was carried out within a frequency range of 125–8,000 Hz, starting at 1,000 Hz. The patient was asked to indicate whether their tinnitus frequency was lower or higher than the sound stimulus played. The frequency was adjusted until the patient identified the most accurate match. Following this, using the frequency determined through pitch matching, the intensity of the tinnitus was measured in 1 dB increments. The pitch and loudness of tinnitus were then manually marked on the audiograms.

Speech audiometry was conducted for each case on the same day as the pure-tone audiometric examinations. The word recognition test utilized acoustic and phonetically balanced words suitable for the native Hungarian-speaking population (as established by Gőtze). Before the examination, some words were presented to the patients. The audiologist determined the ideal intensity level by adding 25 dB to the pure-tone averages. Patients were then asked to indicate whether this intensity level was acceptable. Subsequently, 20 words were presented, and the number of correct responses was recorded, with an additional +5% awarded for each correct answer. This method allowed for the calculation of the maximum percentage of speech understanding. If the maximum score was below 70%, the test was repeated at higher intensities. The intensity was increased until the word recognition percentage improved, ensuring that it never reached the loudness discomfort level. The maximum percentage of word speech recognition was indicated on the audiograms at specific intensity levels, with notations for discrimination loss and rollover.

### Self-reported tinnitus severity measurement

Self-reported tinnitus severity was assessed using the Tinnitus Handicap Inventory (THI), which has been previously validated in Hungarian (14). The THI evaluates the impact of tinnitus on daily functioning through three categories of questions: functional, emotional, and catastrophic. The functional category includes inquiries about daily activities, such as social interactions, household tasks, and stress management. The emotional category addresses issues related to anxiety, depression, and frustration. Meanwhile, the catastrophic category focuses on experiences of serious illness or feelings of lack of control. Patients can respond to each question with 4 points for “yes” and 2 points for “sometimes”. The total THI score is calculated by summing the points from each category. Based on the THI results, five levels of tinnitus handicap can be defined: no handicap (0–16 points), mild (18–36 points), moderate (38–56 points), severe (58–76 points), and catastrophic (78–100 points).

### Statistical analysis

All statistical analyses were performed using IBM SPSS version 25 software (IBM Corporation, Armonk, NY, United States). The normality of the data was assessed using the Shapiro–Wilk test, which indicated that the distribution was not normal. Therefore, the Mann–Whitney *U* test, a non-parametric method, was utilized. The Chi-squared test was employed to analyze categorical data. Continuous variables were reported as medians with interquartile ranges (IQRs). Correlations between the parameters were evaluated using Spearman’s correlation test. A significance level of *p* < 0.05 was established.

## Results

The participants’ basic parameters are reported in Table 1.

**Figure 1:**
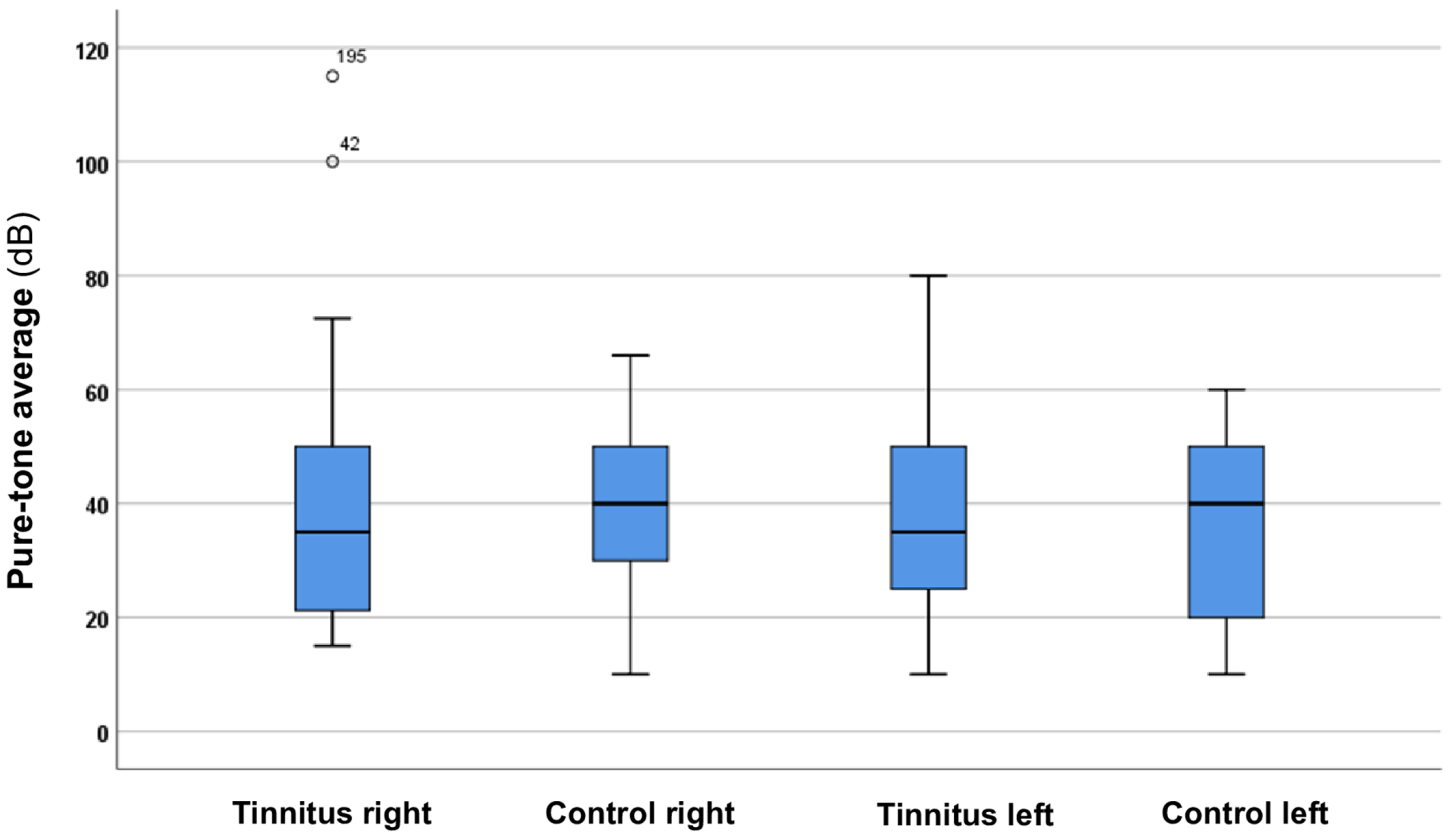
Boxplot displaying the pure-tone averages for both the tinnitus and control groups. The boxes represent the middle 50% of the data, while the whiskers indicate the upper and lower 25%. The black line that divides each box represents the median values. The circles and numbers depict the outliers. Statistical differences were analyzed using the Mann–Whitney *U* test (*p* < 0.05).

Table 1 shows that there are no statistically significant differences in the basic parameters between the study and control groups. Thus, both groups are suitable for further analysis. The pure-tone averages also did not differ statistically between the two groups (see Figure 1). In terms of sex distribution, there is a slight predominance of females in both groups. Most participants report tinnitus symptoms that are either left-sided or bilateral. Based on the onset of tinnitus, it can be inferred that chronic symptoms are predominantly present.

**Figure 2:**
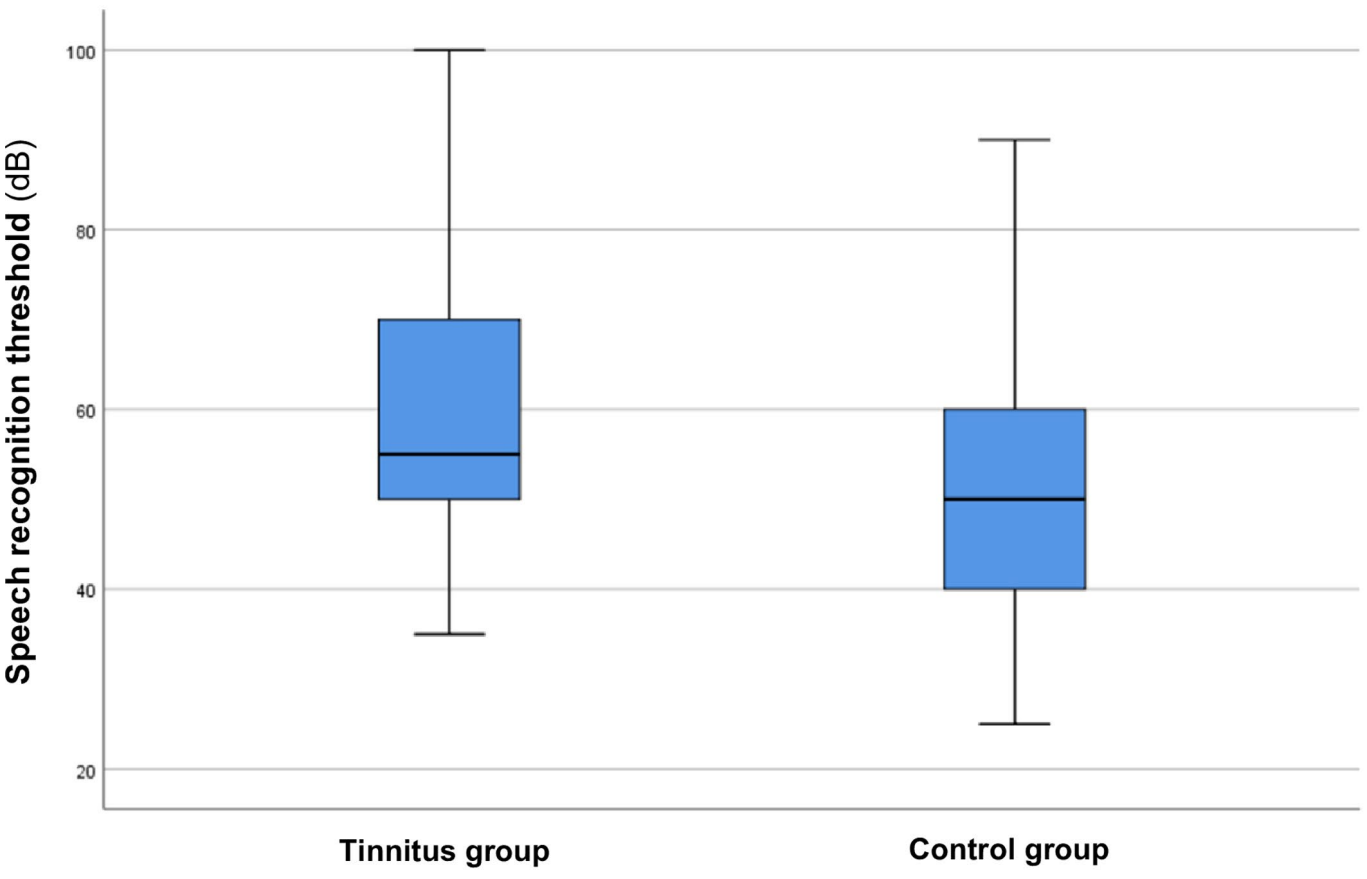
Speech audiometry intensity in the tinnitus and control groups. The boxes represent the middle 50% of the data, while the whiskers indicate the upper and lower 25%. The black line that divides each box represents the median values. Statistical differences were analyzed using the Mann–Whitney *U* test (*p* < 0.05).

In comparing the speech audiometry intensity, patients with tinnitus showed significantly higher values (*p* < 0.00001*, *z*-score: −5.19). This suggests that individuals with tinnitus have poorer speech recognition scores compared to those with sensorineural hearing loss who do not have tinnitus (Figure 2).

**Figure 3:**
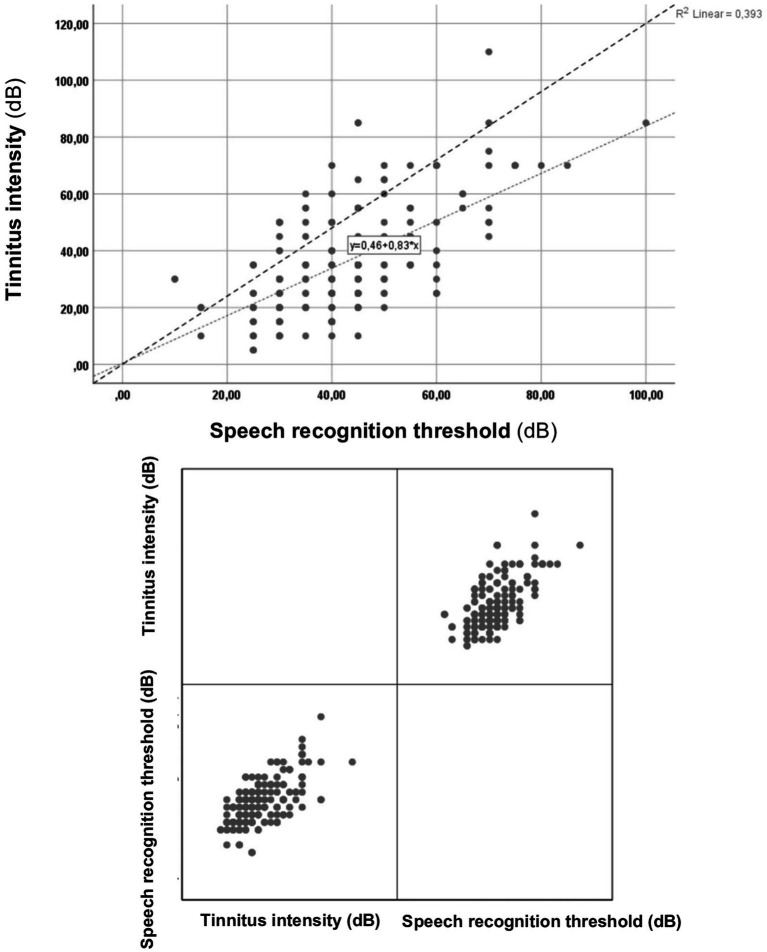
Correlation between tinnitus intensity and speech audiometry intensity.

In the next step of the investigation, the correlations between tinnitus intensities and speech audiometry intensity were analyzed (Figure 3).

In analyzing the correlation between tinnitus intensity and speech audiometry intensity (see Figure 3), a statistically significant (*p* = 0.000*), positive correlation (rho = 0.581) was observed. This finding indicates that as tinnitus intensity increases, higher intensity levels are required in speech audiometry to achieve the highest word recognition score. In other words, tinnitus has a significant impact on speech recognition, leading to poorer scores.

**Figure 4:**
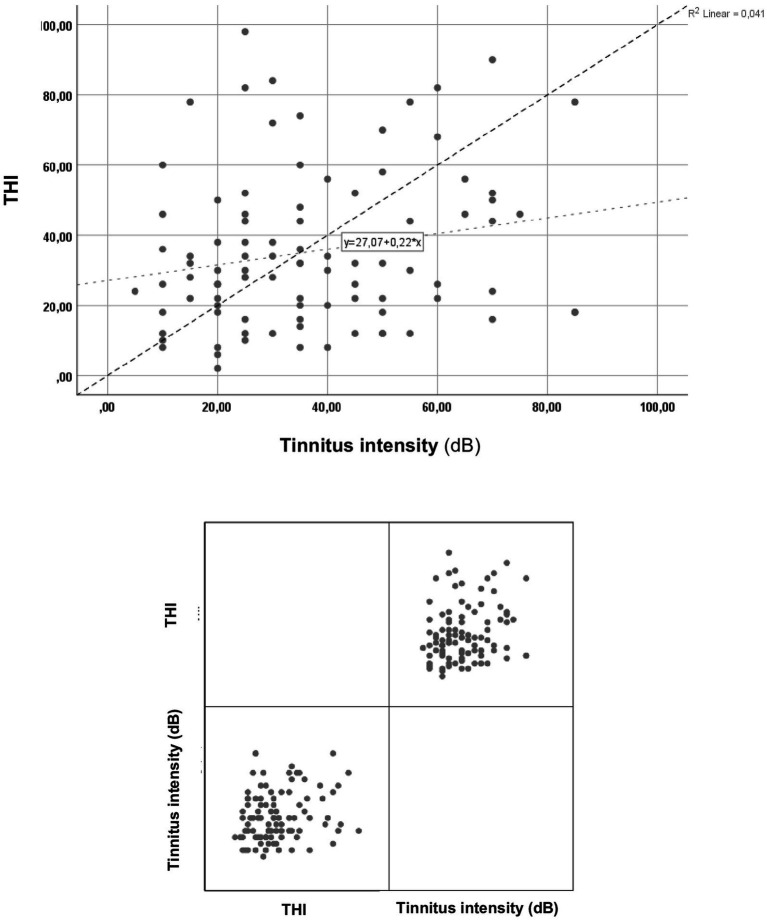
Correlation analysis on tinnitus intensities and total THI scores. THI, tinnitus handicap inventory.

In the next phase, an analysis was performed to explore the potential correlation between self-reported tinnitus severity and tinnitus intensity. The results are shown in Figure 4.

In analyzing the correlation between tinnitus intensity and total THI scores, a statistically significant (*p* = 0.027*), positive correlation (rho = 0.227) was observed. This indicates that higher levels of tinnitus intensity result in significantly greater tinnitus-related handicaps.

## Discussion

This study aimed to analyze the results of speech audiometry in relation to pure-tone averages and tinnitus intensities. A key finding of this investigation was that there was a significant correlation between tinnitus intensity levels and speech audiometry intensity. This indicates that more intense tinnitus leads to notably poorer speech comprehension, underlining the impact of tinnitus on daily functioning. Furthermore, higher intensities were required in the tinnitus group to achieve maximal speech comprehension compared to the control group. Additionally, the significant correlation between tinnitus intensities and total THI scores further reinforces how tinnitus intensity affects quality of life.

Research on speech understanding in individuals with tinnitus is relatively sparse. For example, one study involving participants with pure-tone averages exceeding 20 dB found that those with tinnitus had significantly higher speech reception threshold noise (SRT) values compared to a group without tinnitus. Additionally, more severe tinnitus was linked to higher SRT values. These findings indicate that individuals with tinnitus may experience poorer speech intelligibility in noise (15). Another investigation found that only patients with conductive hearing loss and high-frequency tinnitus exhibited lower thresholds in speech audiometry compared to participants without tinnitus (16). However, in the current study, patients with sensorineural hearing loss, regardless of frequency ranges, showed lower speech recognition scores. Another investigation observed that tinnitus pitch did not correlate with the worst consonant recognition. However, sentence-initial word recognition was affected by hearing loss, while patients with tinnitus reported significantly higher error rates in full-sentence recognition (17). Furthermore, a study found that the results of pure-tone audiometry, otoacoustic emission and auditory brainstem response did not differ in young adults with and without tinnitus. However, tinnitus patients demonstrated significantly decreased speech-in-noise reception (18). These results highlight the importance of measuring speech perception in tinnitus. According to an investigation, 74.2% of ears with tinnitus demonstrated good speech perception. However, significantly higher values were observed in the tinnitus group regarding speech audiometry (19). Contrary to our findings, a previous study found that among elderly participants matched by age, cognitive status, and mean pure-tone averages (at 16 dB), tinnitus did not significantly affect speech comprehension. This difference may be attributed to the examination of only elderly individuals; thus, speech comprehension could be more significantly affected by aging rather than tinnitus. Additionally, differences in auditory processing have been identified: individuals showed impaired abilities to detect tones in noise, heightened sensitivity to intensity changes, and greater sensitivity to interaural differences. Furthermore, there were non-significant correlations between the THI and speech comprehension, which aligns with our results. However, it is important to note that during the investigation, participants primarily reported minor difficulties with tinnitus as indicated by the THI. We found no correlation between speech comprehension and the THI, although tinnitus intensity did correlate significantly with the THI. This suggests that the loudness of tinnitus has a considerable impact on quality of life (20).

In everyday clinical practice, many patients have difficulty understanding speech due to the sounds of their tinnitus, even when their pure-tone audiometry results are normal. This indicates that tinnitus and impaired speech comprehension may share a common pathway involving the central auditory system. In a previous investigation, researchers analyzed the effects of tinnitus on auditory spectral and temporal resolution. It was initially assumed that the abilities to resolve auditory spectral and temporal information, as well as speech comprehension, would be similar in individuals with unilateral tinnitus who have normal hearing and those with unilateral or bilateral tinnitus who have hearing loss. However, the study found that participants with tinnitus exhibited worse speech comprehension compared to those without tinnitus. These findings underscore the impact of tinnitus on speech perception, independent of hearing sensitivity (21). Hearing loss severity clearly leads to poorer speech comprehension. However, it has been noted that patients with tinnitus who have normal hearing also report difficulties in understanding speech (22). This study also found that worsening speech comprehension can occur even without hearing loss. Previous reports have emphasized that central inhibitory deficits play a crucial role in the deterioration of speech comprehension in noisy environments for individuals with tinnitus. This mechanism suggests that a heightened central gain occurs due to reduced sensory input from the inner ears, such as hearing loss that may not be detectable through standard pure-tone audiometry. Consequently, this leads to increased spontaneous and stimulus-induced neural activity (22, 23). Furthermore, the central inhibitory deficit hypothesis may also be supported by observations of improved speech comprehension following tinnitus reduction (24). Anatomically, the medial olivary cochlear system, which extends from the auditory cortex to the inner ear and OHCs, is involved in processing interaural time differences, a function that is essential for sound localization in noisy settings. The medial olivocochlear bundle inhibits OHCs by releasing acetylcholine, which affects cochlear amplification and the sensitivity of inner ear hair cells as well as the afferent fibers of the eighth cranial nerve. The functional consequence of the olivocochlear reflex is improved detection and discrimination of sounds in noisy environments (25). This reflex is part of the “top-down” or efferent modulation involved in tinnitus. Previous studies have indicated increased inhibition of the OHCs’ mediated by the medial olivocochlear reflex. However, it remains unclear whether these changes in tinnitus are pathological or serve a protective role (26). In summary, both peripheral and central hearing mechanisms play a role in the decline of speech perception in individuals with tinnitus.

The significant correlation between tinnitus loudness and severity, as measured by the THI, aligns with previous findings. For example, a study demonstrated significant correlations between the THI, visual analogue scale annoyance, and tinnitus loudness (27). Another investigation found a strong correlation between Tinnitus Loudness Scaling and the THI scores (28). These correlations are crucial, as significant relationships were found between self-reported tinnitus burden, quality of life, and depression (29). Tinnitus loudness should be reduced to prevent impairing quality of life and the onset of psychiatric symptoms. A recent investigation emphasized the significant impact of hearing impairment on self-reported tinnitus severity. The findings revealed that individuals with hearing impairment tended to have higher scores on the auditory component of the Tinnitus Functional Index (TFI) questionnaire, as well as higher scores on both the THI questionnaire and its emotional component (30).

Another important aspect of speech comprehension that requires further research is the challenges associated with fitting hearing aids for individuals with tinnitus. During the fitting process, various factors, including aesthetic considerations and subjective perceptions, must be taken into account to maximize speech comprehension (31). Additionally, tinnitus should also be considered as a significant factor. However, it is worth noting that fitting hearing aids for sensorineural hearing loss can effectively reduce the loudness and disturbances associated with tinnitus. For example, a review of hearing aids for tinnitus reinforced the advantages of fitting hearing aids for tinnitus management. This emphasizes the necessity of incorporating hearing aid fitting aspects in future tinnitus studies (32).

While this investigation has its strengths, there are also some limitations that need to be addressed. First, the cross-sectional design restricts the generalizability of the results. Additionally, speech comprehension was assessed only in a quiet environment, which does not accurately represent real-life situations where noise is often present. Additionally, the central changes in the background of speech comprehension worsening could not be analyzed, nor could objective hearing tests be analyzed. Future studies should address limitations by analyzing speech comprehension more thoroughly, for example, by applying the matrix sentence test (33). However, we believe that our findings enhance the understanding of the mechanisms of tinnitus.

## Conclusion

The notable differences in speech understanding between the tinnitus group and the control group emphasize the significant impact tinnitus has on speech comprehension. Furthermore, the strong connection between tinnitus intensity and speech audiometry intensity reinforces this finding. Additionally, the relationship between tinnitus intensity and THI scores underscores how the loudness of tinnitus affects self-reported severity levels. Therefore, the results of this investigation highlight the need to reduce tinnitus loudness to improve speech comprehension, enhance quality of life, and prevent the development of co-occurring psychiatric symptoms.

## Data Availability

The raw data supporting the conclusions of this article will be made available by the authors, without undue reservation.
